# Open field trial of genetically modified parthenocarpic tomato: seedlessness and fruit quality

**DOI:** 10.1186/1472-6750-5-32

**Published:** 2005-12-21

**Authors:** Giuseppe Leonardo Rotino, Nazareno Acciarri, Emidio Sabatini, Giuseppe Mennella, Roberto Lo Scalzo, Andrea Maestrelli, Barbara Molesini, Tiziana Pandolfini, Jessica Scalzo, Bruno Mezzetti, Angelo Spena

**Affiliations:** 1 CRA – Istituto Sperimentale per l'Orticoltura Sezione di Montanaso Lombardo, Italy; 2 CRA – Istituto Sperimentale per l'Orticoltura Sezione di Monsampolo del Tronto, Italy; 3 CRA – Istituto Sperimentale per l'Orticoltura di Pontecagnano, Italy; 4 CRA. – Istituto Sperimentale per la Valorizzazione Tecnologica dei Prodotti Agricoli di Milano, Italy; 5 Dipartimento Scientifico e Tecnologico, Università di Verona, Italy; 6 Dipartimento di Scienze Ambientali e delle Produzioni Vegetali, Università Politecnica delle Marche di Ancona, Italy

## Abstract

**Background:**

Parthenocarpic tomato lines transgenic for the *DefH9-RI-iaaM *gene have been cultivated under open field conditions to address some aspects of the equivalence of genetically modified (GM) fruit in comparison to controls (non-GM).

**Results:**

Under open field cultivation conditions, two tomato lines (UC 82) transgenic for the *DefH9-RI-iaaM *gene produced parthenocarpic fruits. *DefH9-RI-iaaM *fruits were either seedless or contained very few seeds. GM fruit quality, with the exception of a higher β-carotene level, did not show any difference, neither technological (colour, firmness, dry matter, °Brix, pH) nor chemical (titratable acidity, organic acids, lycopene, tomatine, total polyphenols and antioxidant capacity – TEAC), when compared to that of fruits from control line. Highly significant differences in quality traits exist between the tomato F1 commercial hybrid Allflesh and the three UC 82 genotypes tested, regardless of whether or not they are GM. Total yield per plant did not differ between GM and parental line UC 82. Fruit number was increased in GM lines, and GM fruit weight was decreased.

**Conclusion:**

The use in the diet of fruits from a new line or variety introduces much greater changes than the consumption of GM fruits in comparison to its genetic background. Parthenocarpic fruits, produced under open field conditions, contained 10-fold less seeds than control fruits. Thus parthenocarpy caused by *DefH9-RI-iaaM *gene represents also a tool for mitigating GM seeds dispersal in the environment.

## Background

The debate on genetically modified (GM) crop plants has been focused on two main uncertainties: 1) whether a GM plant differs from its non-GM progenitors only in the introduced trait of interest, 2) whether a GM plant is safe in the environment with respect to gene flow and seed dispersal. To address these questions, we have chosen parthenocarpy, the development of the fruit in absence of fertilization, to evaluate the equivalence of GM and non-GM fruit and to evaluate the advantages of parthenocarpy produced by genetic engineering compared to traditional methods. In this work, we present an analysis of parthenocarpic tomato fruit obtained from field-grown GM plants to address some aspects of the equivalence of GM fruit.

The trait of parthenocarpy is particularly important for crop plants whose commercial product is their fruit [1,2]. During flowering, adverse environmental conditions may either prevent or reduce pollination and fertilization decreasing fruit yield and quality. Moreover, parthenocarpic fruits are seedless, and seedlessness is highly valued by consumers in some fruit (e.g. table grape, citrus, eggplant, cucumber).

Parthenocarpic fruits have been produced by traditional breeding methods based either on mutant lines or other strategies such as alteration of the ploidy level as in banana and watermelon [2]. However, genetic parthenocarpy has been used only for a limited number of species and varieties. In some species and varieties, seedless fruit production is often achieved by external application of plant growth regulators as in the case of grape, tomato and eggplant [3].

Several methods to genetically engineer parthenocarpic fruit development have been proposed, and some have also been tested experimentally in crop plants [1,2]. Thus, transgenic parthenocarpic plants have been obtained for horticultural crops [4-7]. In particular, the chimeric gene *DefH9-iaaM *has been used to drive parthenocarpic fruit development in several species belonging to different plant families [4,5,7]. The *DefH9-iaaM *transgene promotes the synthesis of auxin (IAA) specifically in the placenta, ovules and tissues derived therefrom [4,8]. The agronomical advantages of *DefH9-iaaM *GM plants have been assessed by greenhouse and field trials of *DefH9-iaaM *eggplant [9,10], strawberry and raspberry [7].

The *DefH9-RI-iaaM *gene construct produces high quality parthenocarpic fruits in the tomato cultivar UC 82, a variety used by the processing industry. The *DefH9-iaaM *gene construct gave rise to malformed parthenocarpic fruits because of high sensitivity to auxin that is present in the UC 82 genetic background [8]. The *DefH9-RI-iaaM *gene version is less efficiently translated and has a weaker action in producing IAA than *DefH9-iaaM*, so its use avoids the malformation of the fruit.

Parthenocarpy represents a useful trait in tomato fruit used for industrial purpose as well. This because parthenocarpic fruits are usually either seedless or contain significantly fewer seeds than non-parthenocarpic varieties. Manipulation of fruit and seed quality, size and number has been recently included among the third-generation traits of GM crop plants [11]. In the tomato sauce industry, seed content is a problem and seeds are removed to obtain sauce of good quality. As far as fruit quality concerns, parthenocarpy may also improve fruit quality through increases in the solid soluble content of the fruit [12]. Productivity may also be increased in some seasons because fruit set and fruit growth are less affected under environmental conditions adverse for pollination and/or fertilization including heavy rain, high humidity, hot and dry wind etc.

Processing tomatoes represent the greatest proportion of tomato production (approximately 113 million tons in 2003 [FAO]). In this paper, data on agronomic performance under open field conditions, technological and biochemical characteristics of two *DefH9-RI-iaaM *UC 82 tomato lines are presented and compared to untransformed control. We also included in our analysis a modern F1 hybrid tomato, used by the processing industry, to determine the extent to which biochemical and agronomical parameters vary between different non-GM tomato lines. The data indicate that the use in the diet of fruits from a new line or variety introduces much greater changes than the consumption of GM fruits in comparison to its genetic background.

## Results

**Figure 1:**
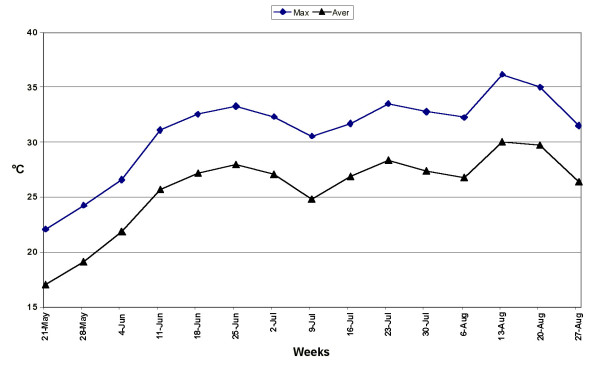
Mean weekly values of maximum and average temperatures from 14 May until 27 August 2003.

The field trial was performed in 2003. The mean air temperature from the first week of June until the end of the growing season was unusually very high and constantly above 25°C and 30°C average and maximum temperature, respectively (Fig. 1).

Fruit production, as measured by marketable fruit yield, was highest in the modern F1 tomato hybrid Allflesh (Table 1). The transgenic parthenocarpic lines Ri4 and Ri5 gave a fruit yield similar to that of the untransformed control UC 82. However, one transgenic parthenocarpic line (Ri4) gave a marketable fruit production that was not statistically different from all the other three genotypes (Ri5, UC 82 and Allflesh).

The two parthenocarpic lines produced a higher number of fruits with respect to the untransformed control UC 82 (Table 1). The increased fruit number per plant was most likely due to an improved fruit set of GM parthenocarpic plants compared to the untransformed control (UC 82). The two parthenocarpic lines produced fruits of smaller size (Table 1). The reduction of fruit weight observed in the two parthenocarpic lines is most likely due to the increased number of fruits per parthenocarpic plant. The hybrid Allflesh gave a number of fruits that was not different from the numbers of both transgenic lines and of the cultivar UC 82.

The unmarketable yield, represented by green and rotten fruits, was not different between the four genotypes (Table 1).

**Figure 2:**
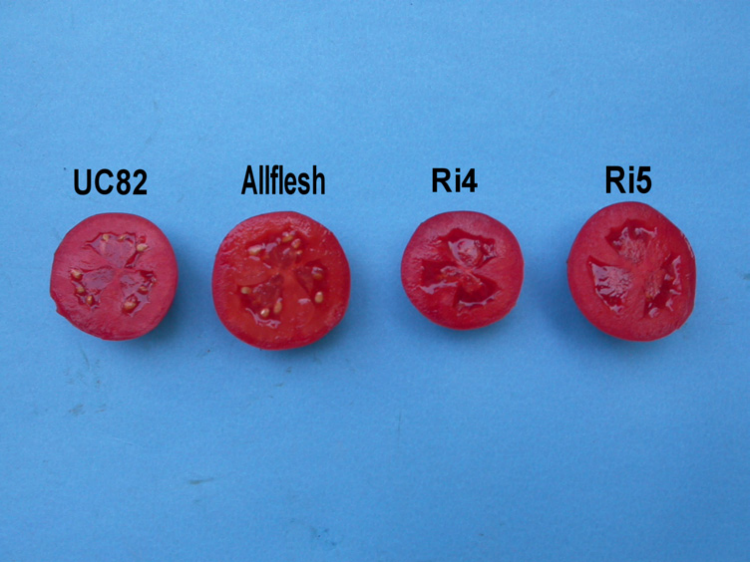
Cut tomato fruits of the four genotypes used: UC 82, Allflesh and GM UC 82 lines Ri4 and Ri5. GM fruits (Ri4 and Ri5) are seedless. UC 82 and Allflesh fruits contain seeds.

The shape of the tomatoes, the puffiness fruit index and the number of locules per fruit did not differ between the genotypes tested (Table 2). The two transgenic lines Ri4 and Ri5 produced a significantly lower percentage of seeded fruit and a significantly reduced number of seeds per fruit compared to the untransformed control and the modern F1 hybrid Allflesh (Table 2 and Fig. 2). This shows that the fruits obtained from the transgenic lines were parthenocarpic.

Colour evaluation of fruits showed that L* values (an index of brightness) did not vary among UC 82 genotypes. A significant higher a* values, representing the red component, was found in non-transformed fruits compared to that of the transgenic fruits (Table 3). The b* value (an index of the yellow colour) was different from the UC 82 control only in Ri4 GM line. °Brix values showed a significant higher soluble sugar content in Allflesh tomatoes (5.3). The °Brix value of Ri5 (4.2) did not differ from UC 82 (3.8), while the °Brix value (4.5) of Ri4 was higher than UC 82. However, the °Brix values of the two transgenic lines were not significantly different. The pH values were close to 4.0 in both the cultivar UC 82 and in the transgenic lines derived from it. Allflesh had a slightly higher pH statistically different from all the other genotypes tested (Table 3). Total acidity values showed no significant variation between the genotypes analyzed. The resistance of skin (fruit firmness) was about 0.4 Kg for all samples. Dry matter content was significantly higher in Allflesh with respect to all three UC 82 genotypes tested (Table 3).

Analysis of the citric, tartaric and oxalic acid amounts showed that citric acid was the most abundant (Table 4). Citric acid content was significantly higher in Allflesh compared to cv. UC 82. The two lines Ri4 and Ri5 had intermediate amounts not significantly different from each other, although Ri4 had a higher content than UC 82. Tartaric acid content did not show any significant difference between the tomatoes produced by the four genotypes tested. Oxalic acid content differed only in transgenic line Ri5. β-carotene content was significantly higher in the tomatoes of the two transgenic parthenocarpic lines, followed by UC 82 and Allflesh. Vitamin C, lycopene and tomatine did not show any significant difference between the four genotypes tested.

The total antioxidant capacity was rather similar in all four genotypes with Allflesh having the highest value which differed significantly only from that of Ri4 line (Table 5). The difference in the total antioxidant activity was attributable to the hydrophilic phase. In fact, hydrophilic and total TEAC were similar both as values and as trend, whereas the antioxidant activity of the lipophilic matrix showed no significant difference between the four genotypes tested and with values so low that they barely contribute to the overall antioxidant capacity. The content of total polyphenols was not significantly different.

In conclusion, tomatoes (UC 82) genetically modified for parthenocarpy, grown under open field conditions, show a fruit yield per plant identical to their corresponding control but a 10-fold reduction in the seeds content. All other tested parameters, with the exception of β-carotene, did not show relevant changes between GM and not GM UC 82 tomatoes.

## Discussion

The open field trial of two transgenic tomato lines obtained from the processing cultivar UC 82 showed that the *DefH9-RI-iaaM *gene was able to induce parthenocarpic fruit development under open field cultivation conditions. Most (approximately 75%) of the fruits produced were seedless and, furthermore, seeded fruits contained significantly less amount of seed (on the average the number of seeds per fruit was 60–80% reduced) than both the corresponding untransformed UC 82 control and the modern cultivar F1 Allflesh (Table 2). These results are in accordance with those from field trial of eggplant where most of the transgenic fruits were seedless [10] and confirmed that *iaaM*-induced parthenocarpy might be used as a tool to reduce transgenic seed dispersal without the need to combine it with male sterility. The significantly fewer number of seeds in *DefH9-RI-iaaM *tomato (UC 82) fruits may represent a desirable trait for processing tomatoes because the absence of seeds simplifies industrial activities which normally discard the seeds from the paste.

Under the rather high temperature that occurred during flowering, fruit set and growth, the *DefH9-RI-iaaM *parthenocarpic transgene allowed the production of a significantly larger number of fruits compared to the untransformed cv. UC 82. This indicates, at least under the conditions tested, an improved ability of the *DefH9-RI-iaaM *plants to set fruits. Poor fruit set and dramatic reduction in fruit size has been reported in tomato plants grown at 26°C under controlled conditions [13]. *DefH9-RI-iaaM *parthenocarpic fruits had a shape similar to that of the control and no malformations were observed, confirming the data obtained for T0 plants [8]. The reduction of weight observed in parthenocarpic fruit grown under open field conditions, might be due to the increased number of fruits caused by the improved fruit set of parthenocarpic plants. A different agronomical practice (i.e. watering, fertiliser regime, etc) apt for high fruit-set lines (i.e. *DefH9-Ri-iaaM *GM) might improve fruit weight, and consequently plant productivity.

Except for the colour coordinate a* (red), the two transgenic parthenocarpic lines showed no relevant variation of the technological properties (°Brix, pH, dry matter, total acidity and firmness) compared to the untransformed control cv. UC 82. Interestingly, all three genotypes in the UC 82 background differed from the modern F1 hybrid Allflesh for the solid soluble content (°Brix value), which is a very important trait for processing tomato. The presence of the transgene *DefH9-RI-iaaM *showed no significant influence on the amount of the biochemical compounds relevant for tomato processing quality. In fact, the organic acids, vitamin C, lycopene and tomatine were not different between the two GM lines and their control. The amount of β-carotene, a beneficial dietary bioactive for humans, was higher in the transgenic lines compared to both the control and the F1 Allflesh. This represents the main difference between transgenic and control fruits. Some natural parthenocarpic variants of tomato have a higher β-carotene level than their original cultivars [12]. No differences were observed for the antioxidant activity and polyphenol content between the transgenic lines and the cv. UC 82. For these traits, few differences were observed between the F1 Allflesh and one UC 82 genotype (Ri4 transgenic). Similar results have been obtained in transgenic tomatoes engineered for other traits [14-16].

To determine the substantial equivalence and identify possible unintended effect in engineered food it has been proposed that biochemical fingerprinting be expanded beyond the comparison between transgenic genotypes and its correspondent untransformed controls to include several non-transgenic lines [17]. This approach can allow the determination of whether any difference originate from metabolic effect associated with the transgenic trait or from expected genetic and/or physiological variation within the species. In the present study the extent of variation was higher between the traditional UC 82 cultivar (transgenic or not) and the last generation F1 hybrid Allflesh.

## Conclusion

This trial has demonstrated that, under open field conditions allowing pollination/fertilization, the *DefH9-Ri-iaaM *transgene was able to sustain parthenocarpic fruit development in the cv. UC 82. Biochemical and technological analyses performed on tomato fruit (GM and non-GM) showed a very little variation that is well within the variability of the species *Lycopersicon esculentum*. Yield per plant did not differ between GM and non-GM, in GM lines fruit weight was decreased, whilst fruit number was increased.

## Methods

### Plant material

The selfed progenies of two single copy transgenic tomato (cv. UC 82) lines carrying *DefH9-RI-iaaM *(named Ri4 and Ri5), the untransformed control cultivar UC 82 and the commercial F1 hybrid "Allflesh 1000" (Peotec) were compared. The Ri4 and Ri5 selfed progenies were selected for kanamycin resistance. Consequently, they were either homozygous or hemizygous. The field trial was performed, following authorization by the Italian Ministry of Health (B/IT/02/10 Pomodoro – *DefH9-iaaM*), at the experimental farm of the Marche Polytechnic University located in Agugliano (Ancona – IT). The experimental design used a latin square with 4 replications, each containing 20 plants at a density of 3 plants/m^2^. The plants were grown in a single row in soil mulched with black plastic polyethylene film (0.05 mm thick) and standard agronomical techniques were applied throughout the growing season. Plantlets at the third-fourth true leaf were transplanted on May 12, 2003. Harvest was in the last week of August. The following traits were recorded: number and weight of ripe tomatoes (marketable production), number and weight of unripe and rotten fruits (unmarketable production). For each plot, the percentage of fruits containing at least 1 seed, the mean number of seeds/fruit, the fruit shape index (polar/equatorial ratio), puffiness index evaluated by an arbitrary scale from 1 (no puffy fruit) to 3 (deep puffy fruit) and the number of locules was recorded using a representative sample of fruits.

### Biochemical and physical analyses

All the analyses were performed on a representative sample of fruits from each replicated plot. The physical quality traits (texture, color, firmness, dry matter content), chemical quality traits (soluble solid content-°Brix, pH, titratable acidity, acidic profile, vitamin C levels, total polyphenol levels) and antioxidant activity were determined after 72 hours from the harvest, with 24 hours conditioning at 4°C prior to analysis.

### Physical analyses

Colour measurements were performed with a reflectance colorimeter Minolta Chromameter CR 200 (Minolta Co., Osaka, Japan), which measured the central part of the surface of tomatoes. L*, a* and b* values were calculated. L* represents the brightness, in particular a value close to 100 means a very high light; a* is an index of red colour (i.e. a high positive value means a strong red colour while a high negative value means a green colour); b* is an index of yellow colour (i.e. a high positive value indicates intense yellow colour while a high negative value indicate a blue colour).

The firmness of the tomato fruits was determined with a dynamometer Instron model 4301 (Instron Corporation, Canton, MA, USA) by measuring the maximum force (kg) required to make an hole on the skin of the tomato using a 1 mm diameter, cylindrical probe pressed into the pulp of each fruit at a speed of 0.1 m/min.

### Chemical analyses

All chemical assays were once replicated within the same sample. Tomatoes were cut and peeled and immediately homogenized by a Waring blender.

Dry matter and pH determinations were performed as reported in [18]. An aliquot of the homogenized tomato tissues was used to measure the soluble solid content (SSC) with a BS model RFM 81 refractometer.

About 10 g of homogenized flesh was used for the determination of titratable acidity and acidic profile. Titratable acidity was determined according to AOAC methods (AOAC. 1980. Official Methods of Analysis, 13^th ^ed. N° 46024 and N° 22061. Association of Official Analytical Chemists, Washington. DC).

The Total Phenolic Content was determined in tomato extracts by the Folin-Ciocalteu based method [19], using gallic acid (GA) as a standard for the calibration curve. Results were calculated as gallic acid equivalent (GAE) in fresh fruit (mg g^-1^). A calibration curve (0-500 mg l^-1 ^of GA) was made prior to the determinations. Samples were read in a KONTRON Uvikon 941 Plus spectrophotometer.

Citric, tartaric and oxalic organic acids were determined from an aqueous extract of the homogenized pulp (10 g plus 20 mL H_2_O), homogenized for 30 seconds with an Ultra-Turrax, then centrifuged at 5600 × *g *for 20 min, and filtered through 0.45 μm filter. The extracts were analyzed by HPLC at 20°C, using 0.02 M H_3_PO_4 _as mobile phase (flow rate: 0.6 mL/min) on an Inertsil ODS-3 column of 0.46 × 25 cm dimension with 5 μm of particle diameter, using detection by a UV-VIS spectrophotometer set at 210 nm. Retention times of standards were: tartaric acid 8.9 min, oxalic acid 9.8 min, citric acid 21.9 min.

Ascorbic acid (vitamin C) was determined from an aqueous extract of the homogenized pulp (10 g plus 20 mL metaphosphoric acid 6% in H_2_O), homogenized for 30 seconds in an Ultra-Turrax, then centrifuged at 5600 × *g *for 20 min, and filtered through a 0.45 μm filter. The extracts were analyzed by HPLC at 20°C, using 0.02 M H_3_PO_4 _as mobile phase (flow rate: 0.6 mL/min) on an Inertsil ODS-3 column of 0.46 × 25 cm dimension with 5 μm of particle diameter detected by a UV-VIS spectrophotometer set at 254 nm. Under these conditions, ascorbic acid has a retention time of 8.6 minutes.

Compounds were identified following HPLC by comparing their retention times with those of commercial standards. Compounds were quantified by plotting different peak areas against concentrations. Results were expressed as mg/100 g dry weight of whole tomato fruits.

The determinations of tomatine, β-carotene and lycopene were performed on tomato fruit samples frozen in liquid nitrogen at harvest and stored at -80°C. The extraction and HPLC analysis procedures used to determine tomatine levels were as reported in [20]. We employed a Luna C_8 _5 μm (stainless steel, 150 × 4.6 mm i.d.) (Phenomenex, Torrance, CA) reversed phase column, equipped with the appropriate pre-column, for the separation along a mobile phase of water-acetonitrile-methanol-0.1 M ammonium phosphate buffer, pH 3.5 (using phosphoric acid) at a flow rate of 0,7 mL/min. The injection volume was 5 μL for both standards and samples; detection was at 205 nm and each separation lasted 15 min at room temperature. Peak area was used for quantification.

For β-carotene and lycopene analyses, 10 g of tomato edible portions were homogenized with 2 mL of 0.1% butylated hydroxytoluene (BHT) in methanol (v/v) for 3 minutes, 15 mL of HPLC grade isooctane were added, the samples were then mixed and incubated at 4°C for 1 hour; a 5 mL aliquot of isooctane extract was dried on a rotatory evaporator at 45°C and the dry residue dissolved in 1 mL of mobile phase. Quantification of compounds was based on the response of commercially available standards treated in the same way as the samples by plotting peak areas against carotenoid concentrations in μg per mL.

Twenty microliters of each sample and standard were filtered through a 0.22 μm Millipore filter and injected on a Partisil 5 ODS-3 (stainless steel, 250 × 4,6 mm i.d.) C_18 _reversed phase column (Whatman) for HPLC analysis. A reversed phase pre-column (Whatman) was also used. Isocratic elution was carried out at 40°C along with a mobile phase of acetonitrile-water-ethyl acetate-ethyl acetate:tetraydrofuran 1:1 (42:10:18:30) containing 0.2% glacial acetic acid. The flow rate was 0,8 mL/min, with detection at 502 nm. Each separation lasted 25 min.

Antioxidant activity (AA) was evaluated according to the TEAC (Trolox equivalent antioxidant capacity), modified assay [21,22]. ABTS, a chromogen colorless substance, is changed into its colored monocationic radical form (ABTS^·+^) by oxidative agents. The absorption peak of ABTS^·+ ^is 734 nm. Addition of antioxidants reduces the ABTS^·+ ^into the colorless form. AA was expressed as μmoles Trolox (an analogue of vitamin E) equivalents g l^-1 ^of fresh weight. To assess the antioxidant status of the fruit, two types of extraction (hydrophilic and lipophilic) were carried out. Extraction of the hydrophilic phase (HE) was performed with a T25 Ultra-turrax blender with frozen fruit samples using an ethanol/water (80/20) extraction solution to a final ratio of 1:10 (w/v). Homogenate was centrifuged (2000 × *g *for 10 min) and the supernatant (HE extract) transferred to vials and stored (-20°C) until assayed. Three extracts were collected per sample. Extraction of the lipophilic phase (LE) was made on pellet by adding acetone (1:4 w/v), and centrifuging at 2000 × *g *for 15 min. The supernatant (LE extract) transferred to vials and stored (-20°C) until assayed. Three extracts were collected for each sample.

Each extract (HE and LE) was recovered and the antioxidant activities were measured separately by recording the absorbance at 734 nm in a spectrophotometer (KONTRON Uvikon 941 Plus). Data are expressed as AA induced by the hydrophilic and lipophilic components, and total antioxidant activity by the sum of the two phases

### Statistical analysis

All the data were subjected to ANOVA according on a Latin square scheme with 4 replicates, Duncan test (p < 0.05) was used for comparison of means.

## Authors' contributions

GLR and NA selected the genetic material and coordinated the field trial; ES and BM were concerned with agronomical work at the field trial; GM, RLS, AM, JS, BM have performed biochemical and technological analysis; NA and ES have done the statistical analysis; GLR wrote the manuscript; BM, TP and AS performed the molecular analysis of the plants and the gene constructs used.

## Figures and Tables

**Table 1:** Mean values ( ± SE) of marketable and unmarketable yields per plant, number of fruits per plant, and fruit weight for two transgenic parthenocarpic lines (Ri4 and Ri5), the untransformed control (UC 82) and the commercial F1 cultivar Allflesh.

Genotype	Marketable yield			Unmarketable yield		
		
	Yield/Plant (g)	N° of fruits	Fruit weight (g)	Green fruits		Rotten fruit
					
				Yield/Plant (g)	N° of fruits	Yield/Plant (g)
		
Allflesh	1906 ± 362 a	32.3 ± 6.1 ab	60.5 ± 3.4 a	98 ± 22 a	3.4 ± 0.5 a	3.8 ± 0.6 a
UC 82	1380 ± 301 b	21.6 ± 4.3 b	65.5 ± 2.5 a	196 ± 65 a	6.2 ± 1.9 a	5.2 ± 1.2 a
Ri4	1538 ± 159 ab	43.2 ± 5.0 a	37.0 ± 1.1 b	123 ± 15 a	5.6 ± 0.6 a	6.9 ± 1.4 a
Ri5	1227 ± 147 b	33.7 ± 3.6 a	38.0 ± 1.5 b	149 ± 44 a	6.7 ± 1.5 a	5.2 ± 0.9 a

For each trait at least one common letter indicates no significant difference according to the Duncan test (α = 0.05).

**Table 2:** Mean values ( ± SE) of fruit shape index (polar/equatorial ratio), puffiness index (1–3), number of locules, percentage of fruits with seeds and number of seeds per fruit in the transgenic parthenocarpic lines (Ri4 and Ri5), the untransformed control (UC 82) and the commercial F1 cultivar Allflesh.

Genotype	Shape index	Puffiness index	N° of locules	Fruits with seeds (%)	N° of seeds/fruit
Allflesh	1.27 ± 0.02 a	1.20 ± 0.03 a	2.36 ± 0.03 a	86.7 ± 5.4 a	68.8 ± 6.2 a
UC 82	1.25 ± 0.02 a	1.85 ± 0.16 a	2.47 ± 0.05 a	85.0 ± 8.8 a	36.5 ± 6.5 b
Ri4	1.24 ± 0.03 a	1.43 ± 0.12 a	2.38 ± 0.04 a	26.7 ± 5.4 b	18.4 ± 3.8 c
Ri5	1.27 ± 0.03 a	1.62 ± 0.13 a	2.45 ± 0.16 a	20.0 ± 4.7 b	11.4 ± 3.8 c

For each trait at least one common letter indicates no significant difference according to the Duncan test (α = 0.05).

**Table 3:** Mean values ( ± SE) of colour (coordinate L*, a* and b*), °Brix, dry matter (DM), titratable acidity (mEq/100 mL NaOH 0,1 N) and skin resistance (firmness) detected in the four genotypes tested.

Genotypes	Colour L*	a*	b*	°Brix	pH	DM (%)	Titr. ac.	Firmness (kg)
						
Allflesh	39.2 ± 0.37 b	36.5 ± 0.54 a	25.4 ± 0.48 c	5.3 ± 0.18 a	4.29 ± 0.06 a	6.19 ± 0.18 a	6.12 ± 0.12 a	0.43 ± 0.03 a
UC 82	41.1 ± 0.19 a	35.8 ± 0.12 a	27.9 ± 0.35 a	3.8 ± 0.14 c	4.08 ± 0.04 bc	5.06 ± 0.22 bc	7.23 ± 0.8 a	0.40 ± 0.02 a
Ri4	39.9 ± 0.34 ab	34.1 ± 0.09 b	25.9 ± 1.22 bc	4.5 ± 0.12 b	3.96 ± 0.01 c	4.85 ± 0.01 c	6.12 ± 0.11 a	0.43 ± 0.02 a
Ri5	40.0 ± 0.59 ab	34.0 ± 0.44 b	27.1 ± 0.42 ab	4.2 ± 0.23 bc	4.11 ± 0.01 b	5.24 ± 0.18 b	6.70 ± 0.54 a	0.41 ± 0.03 a

For each trait at least one common letter indicates no significant difference according to the Duncan test (α = 0.05).

L* represents the brightness; a* is an index of red colour (i.e. higher the value, stronger the red colour); b* is an index of yellow colour (i.e. higher the value, more intense the yellow colour).

**Table 4:** Mean values ( ± SE) of citric acid, tartaric acid, oxalic acid, vitamin C (mg/100 g d.w.), β-carotene and lycopene (μg g^-1 ^d.w.), and tomatine (mg g^-1 ^d.w.) detected in the four genotypes tested.

Genotypes	Citric	Tartaric	Oxalic	Vit C	β-carotene	Lycopene	Tomatine
Allflesh	2194 ± 470 a	40.18 ± 1.4 a	37.30 ± 4.5 a	539.6 ± 44.6 a	491.1 ± 19.7 c	659.5 ± 55.1 a	189.4 ± 34.5 a
UC 82	1434 ± 122 b	32.06 ± 5.8 a	35.97 ± 2.5 a	445.6 ± 69.7 a	566.6 ± 8.6 b	815.9 ± 36.7 a	248.2 ± 52.2 a
Ri4	2271 ± 209 a	30.39 ± 1.7 a	36.03 ± 5.0 a	436.5 ± 59.2 a	698.9 ± 10.1 a	928.5 ± 113 a	202.8 ± 33.9 a
Ri5	1837 ± 419 ab	40.58 ± 3.2 a	22.95 ± 4.5 b	374.3 ± 43.5 a	643.6 ± 15.1 a	811.1 ± 34.1 a	169.3 ± 45.4 a

For each trait at least one common letter indicates no significant difference according to the Duncan test (α = 0.05).

**Table 5:** Mean values ( ± SE) of total trolox equivalent antioxidant capacity (TEAC-μmol g^-1 ^f.w.), hydrophilic and lypophilic phases, and total polyphenols (mg gallic acid g^-1 ^f.w.) detected in the four genotypes tested.

Genotypes	TEAC			Total Polyphenols
		
	Total	Hydrophilic	Lypophilic	
		
Allflesh	3.01 ± 0.08 a	2.60 ± 0.07 a	0.42 ± 0.02 a	9.03 ± 1.01 a
UC 82	2.79 ± 0.14 ab	2.40 ± 0.06 ab	0.40 ± 0.08 a	7.55 ± 0.34 a
Ri4	2.50 ± 0.07 b	2.11 ± 0.02 c	0.39 ± 0.05 a	9.32 ± 0.37 a
Ri5	2.71 ± 0.07 ab	2.29 ± 0.07 bc	0.43 ± 0.02 a	7.78 ± 0.19 a

For each trait at least one common letter indicates no significant difference according to the Duncan test (α = 0.05).
